# Supplementary material for: The interplay between supercoiling and DNA modifying enzymes at the single-molecule level
Source: Sci Rep. 2026 May 13;16:21964. doi: 10.1038/s41598-026-52713-2 (PMC13365190; doi:10.1038/s41598-026-52713-2)
Supplement: Supplementary file 1 — Supplementary Material 1 [file 41598_2026_52713_MOESM1_ESM.docx]

**The interplay between supercoiling and DNA modifying enzymes at the single-molecule level**

Elise M. Wilkinson^1^, Antoine M. van Oijen^1^, Timothy D. Craggs^2^, Stefan H. Mueller^1*^, and Lisanne M. Spenkelink^1*^

^1^ Molecular Horizons and School of Science, University of Wollongong, Wollongong, NSW, Australia

^2^ Sheffield Institute for Nucleic Acids, Department of Chemistry, University of Sheffield, Sheffield, S3 7HF, United Kingdom

* To whom correspondence should be addressed. Email: lisanne@uow.edu.au

**Supplementary Table S1:** Oligonucleotide sequences used in this study. MultiBioT_cap was annealed to ConstainedCappingCy5 and ConstrainedForkEnd separately to construct two different oligo blocks.

| **Experiment** | **Component** | **Sequence** |
| --- | --- | --- |
| 6-biotin constrained template | MultiBioT_cap | GAT AAC TAC GAT ACG GGA GGG C/iBiodT/T CAG TGC /iBiodT/GC AAT GA/iBiodT/ ACC GCG AGA CCC ACG C/iBiodT/C ACC GGC /iBiodT/CC AGA  TT/iBiodT/ ATC AGC AAT AAA CCA GCA TG |
|  | ConstrainedCappingCy5 | /5Phos/CT GGT TTA TTG /iCy5/CT GAT AAA TCT GGA GCC GGT GAG CGT GGG TCT CGC GGT ATC ATT GCA GCA CTG AAG CCC TCC CGT ATC GTA  GTT ATC |
|  | ConstrainedForkEnd | /5Phos/CG GTC ATG CTG GTT TAT TGC TGA TAA ATC TGG AGC CGG TGA GCG TGG GTC TCG CGG TAT CAT TGC AGC ACT GAA GCC CTC CCG TAT  CGT AGT TAT CCG AT |

**Supplementary Table S2**: EcoRV sequence

|  |  |  |
| --- | --- | --- |
| EcoRV | EcoRV_snap_tag | A CCC GGC ATG ACA GGA GGA TCG AGG CCT CTt aat acg act cac tat agg gga att gtg agc gga taa caa ttc ccc tct aga aat aat TTT GTT TAA CTT TAA GAA GGA GAT ATA CAT ATG GAC AAA GAC TGC GAA ATG AAA CGT ACC ACC CTG GAC TCT CCG CTG GGT AAA CTG GAA CTG TCT GGT TGC GAA CAG GGT CTG CAC GAA ATC ATC TTC CTG GGT AAA GGT ACC TCT GCG GCG GAC GCG GTT GAA GTT CCC GCA CCG GCT GCG GTT CTG GGT GGT CCG GAA CCG CTG ATG CAG GCG ACC GCG TGG CTG AAC GCG TAC TTC CAC CAG CCG GAA GCG ATC GAA GAA TTC CCG GTT CCG GCG CTG CAC CAC CCG GTT TTC CAG CAG GAA TCT TTC ACC CGT CAG GTC TTG TGG AAA CTG CTG AAA GTT GTT AAA TTC GGT GAA GTT ATC TCT TAC TCT CAC CTG GCG GCG CTG GCG GGT AAC CCG GCa GCG ACC GCG GCa GTT AAA ACC GCG CTG TCT GGT AAC CCG GTT CCG ATC CTG ATC CCG TGC CAC CGT GTT GTT AAC ATC AAC GGT ACC GTT GGT GGT TAC GAA GGT GGT CTG GCG GTT AAA GAA TGG CTG CTG GCG CAC GAA GGT CAC CGT CTG GGT AAA CCG GGT CTG GGT ggt acc ggc ggt ctg gtt cca cgt ggc tcc GGA GGC TCT GCG GGT TCT GCA GCT GGT TCT GAA TTC atg tcc ctt cgt agc gat ctg atc aac gcc ctg tat gac gaa aac cag aaa tac gat gtg tgt ggc atc atc agc gcg gaa ggt aaa atc tat ccg ctg ggt agc gat acc aaa gtt ctg agc acc atc ttc gaa ctg ttc tcc cgt ccg atc atc aac aag atc gct gaa aaa cac ggt tat atc gtt gaa gaa cca aaa cag cag aac cac tac cca gac ttt acc ctg tac aag cca tcc gaa ccg aac aaa aaa atc gcc atc gat atc aaa acc act tat acc aac aaa gag aat gaa aaa atc aaa ttc acc ctg ggt ggt tac acc tcg ttc att cgt aac aac act aaa aac atc gtg tac ccg ttt gat cag tac atc gcg cac tgg atc att ggc tac gtg tac act cgt gtt gcc acc cgt aaa tct tcc ctt aaa acc tat aac atc aac gaa ctg aac gaa att ccg aaa ccg tat aaa ggt gta aaa gtg ttt ctg caa gat aaa tgg gtt atc gca ggc gac ctg gcg ggc agc ggt aat aca acg aac atc ggt tcc att cat gcc cac tat aag gac ttt gtg gaa ggt aaa ggc atc ttc gat tct gaa gat gaa ttt ctg gac tac tgg cgc aac tat gag cgt acc agc cag ctg cgt aac gac aaa tat aac aac att tcg gaa tac cgt aac tgg att tat cgt ggt cgc aag GGC GGT GGC AGC GGT GGC GGT AGC GGC GGT GGC AGC GGC ggc ctg aac gat att ttt gaa gcg cag aaa att gaa tgg cat gaa gat tat aaa gat cat gat ggt gat tat aaa gat cac gat atc gac tac aaa gac gat gac gac aag TAA gat ccg aat tcg cgg ccg cta aca aag ccc tcg agg aag ctg agt tgg atc ctg cca ccg ctg agc aat aaC TAG CAT AAC CCC TTG GGG CCT CTA AAC GGG TCT TGA GGG GTT TTT TGG GTC GAG CAC TGA CTT AGA GCG GCC GCC AC |


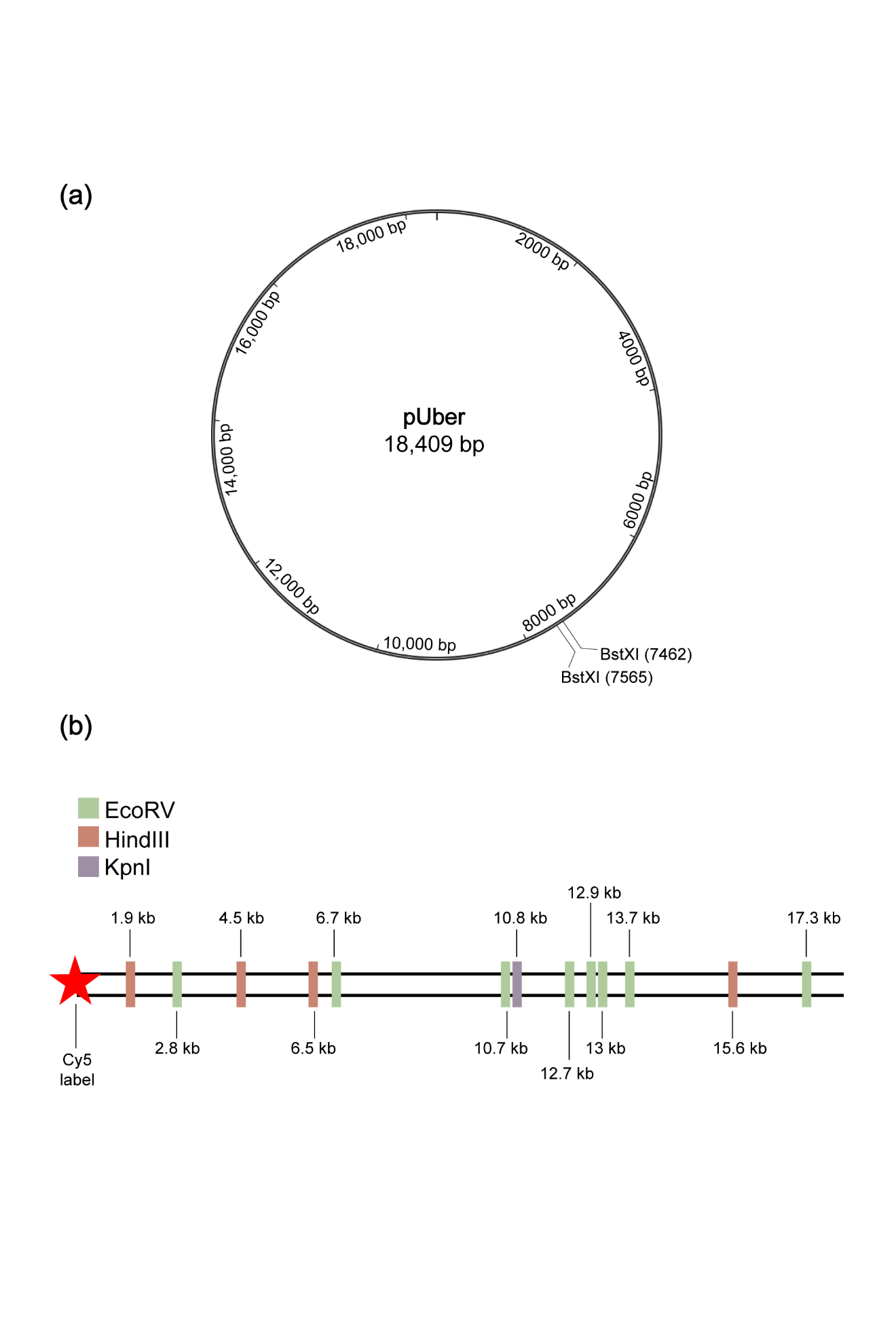


**Supplementary Figure S1:** Plasmid map of pUber and location of EcoRV, HindIII, and KpnI restriction sites. **(A)** Plasmid map of the 18-kb plasmid pUber, highlighting the positions of the BstXI restriction sites used to linearize the plasmid for template DNA template construction. **(B)** Simplified schematic of the constrained DNA template produced from linearized pUber with the positions of the Cy5 end label and restriction sites for EcoRV, HindIII, and KpnI indicated.


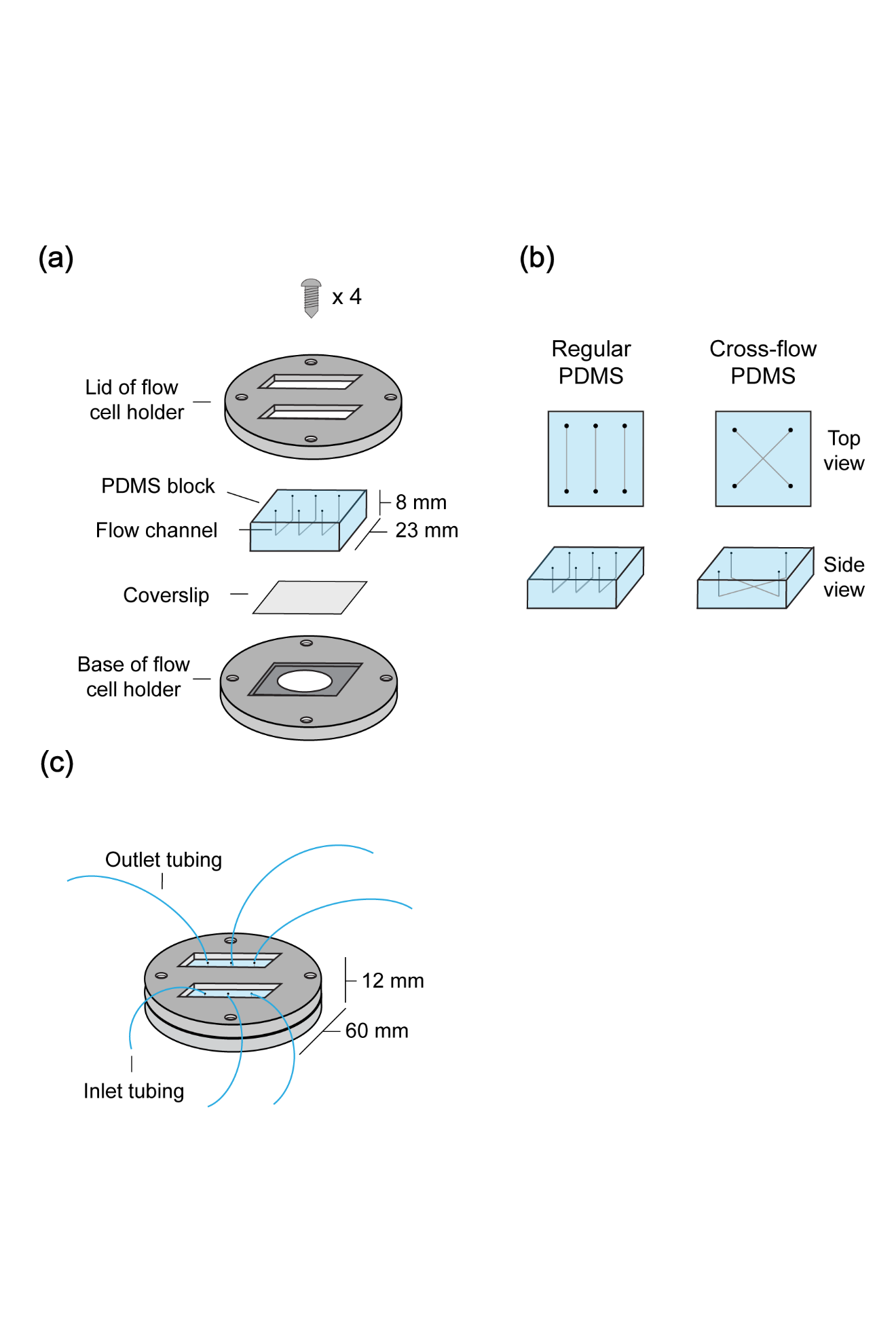


**Supplementary Figure S2:** Flow cell construction. **(A)** Schematic of flow cell components and assembly. The PEG-biotin-functionalized microscope coverslip sits on top of the flow cell holder base. A PDMS block, either three channel or cross-flow, is placed on top of the coverslip. The flow cell holder lid is then secured to the top with 4 screws. **(B)** Schematics of the regular three channel PDMS block and the cross-flow PDMS block, highlighting the channels from top and side views. **(C)** Schematic of constructed flow cell with inlet and outlet tubing inserted.

**
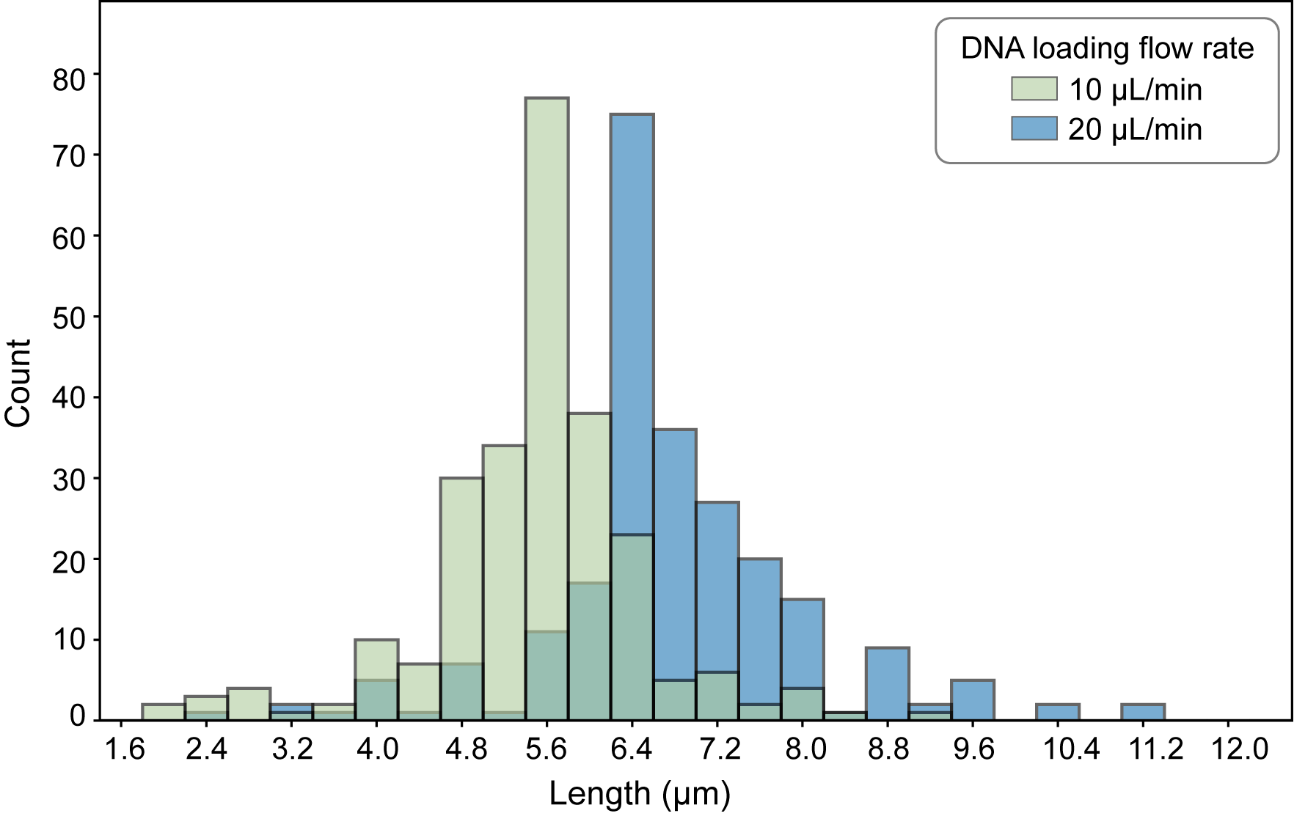
**

**Supplementary Figure S3:** DNA stretch Analysis. Histogram showing distribution of DNA lengths in pixels for two different DNA-loading flow rates, 10 µL/min and 20 µL/min. When DNA is loaded into the flow cell at a flow rate of 10 µL/min, the average length of the DNA templates are 5.5 ± 1.0 µm (n = 241). When DNA is loaded at a flow rate of 20 µL/min, the average length of the DNA templates are 6.8 ± 1.4 µm (n = 250).


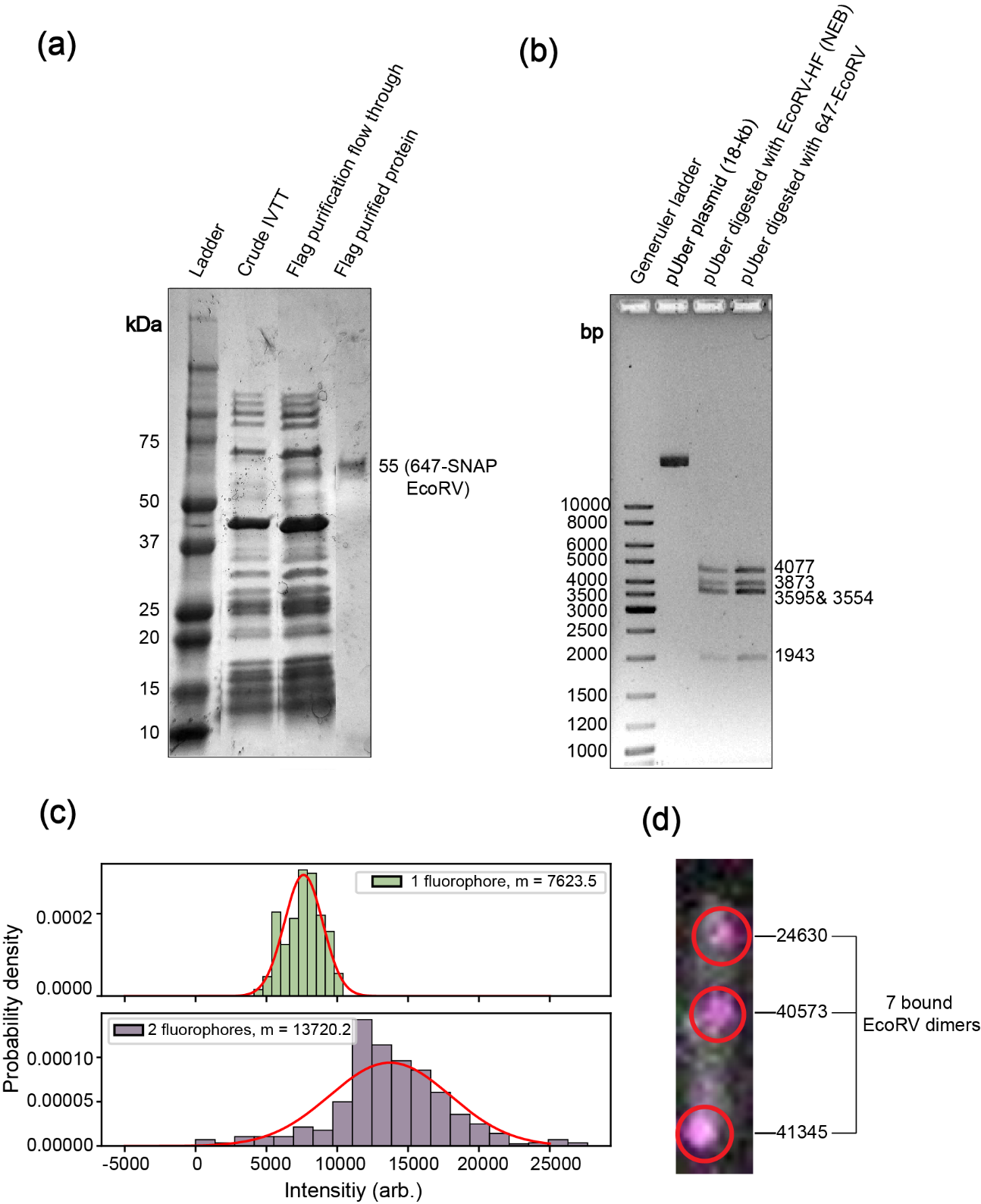


**Supplementary Figure S4:** Cell-free expression and labeling of EcoRV. **(A)** Purification of EcoRV following cell-free expression with IVTT and fluorescent labeling. A 4–20% SDS-PAGE gel showing the sample after IVTT prior to flag purification (lane 2), the flag purification flow through (lane 3), and the sample following flag purification where a single band is present at the expected size of 55 kDa, indicating successful purification (lane 4). **(B)** 647-EcoRV activity test. 1% agarose gel showing the digestion of the 18-kb plasmid pUber with commercial EcoRV-HF (NEB) (lane 3) and 647-EcoRV (lane 4) following labeling and purification. Results indicate that 647-EcoRV is active and efficient. **(C)** Distribution of fluorescence intensities obtained through a photobleaching assay. The average intensity of one fluorophore (EcoRV monomer) is equal to 7623.5, and the average intensity of two fluorophores (EcoRV dimer) is equal to 13720.2. **(D)** Determining the number of EcoRV restriction sites occupied on a single DNA template. Average z-projections of each molecule were generated and the 647-nm signal that was colocalized with a DNA template was manually picked. The integrated intensity of each colocalized 647-nm spot was determined and the intensity for all spots on a single molecule were summed. This value was then divided by the average intensity of two 647 fluorophores, which corresponds to the intensity of an EcoRV dimer. From this, the number of EcoRV restriction sites occupied per molecule was determined.

**
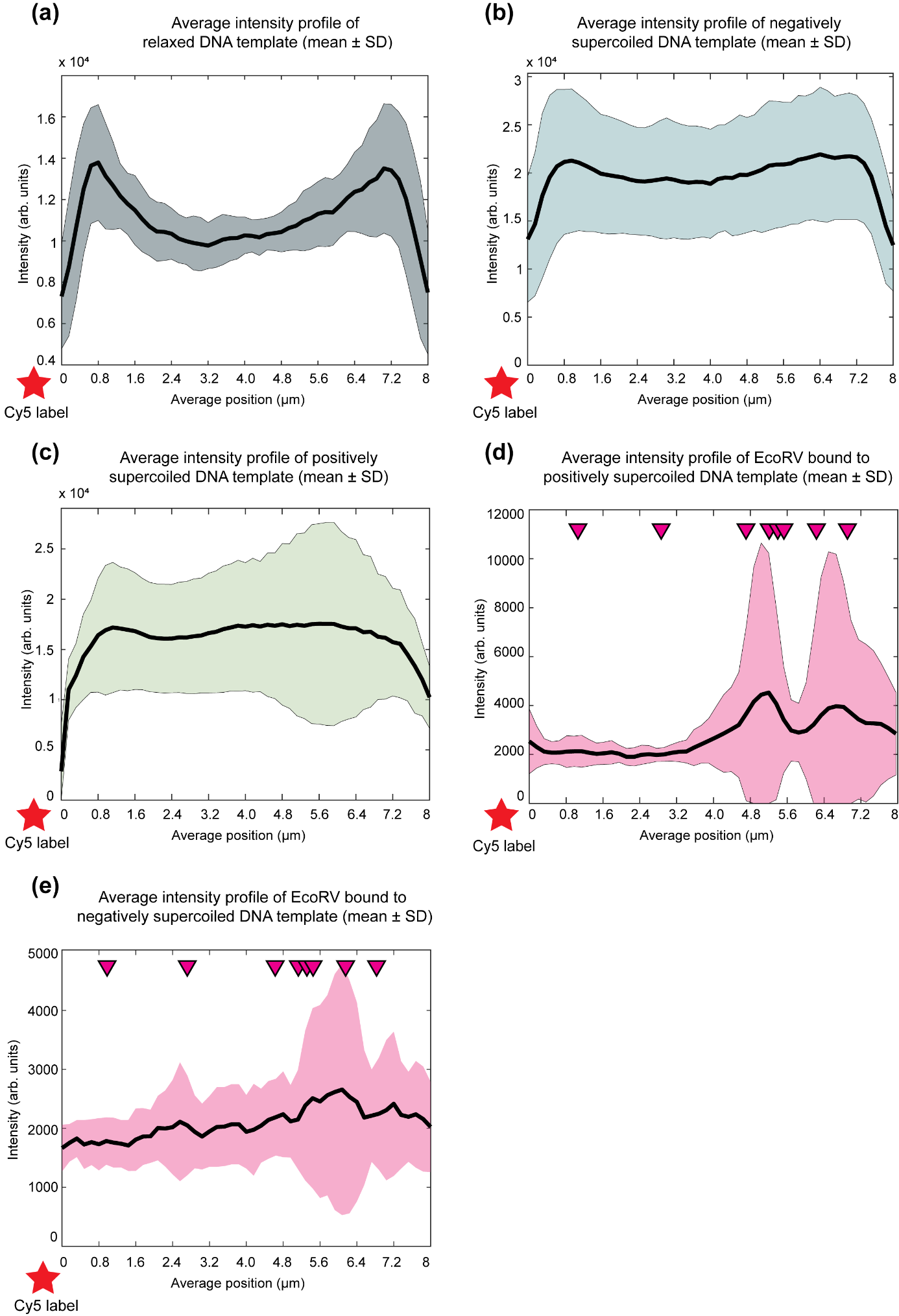
**

**Supplementary Figure S5:** Average intensity profiles of different supercoiled states and bound EcoRV. **(A)** Average intensity profile of relaxed (non-supercoiled) DNA. The position of the Cy5 end label is indicated by a red star. **(B)** Average intensity profile of negatively supercoiled DNA. The position of the Cy5 end label is indicated by a red star. No sequence-dependent plectoneme pinning is observed. **(C)** Average intensity plot of positively supercoiled DNA. The position of the Cy5 end label is indicated by a red star. No sequence-dependent plectoneme pinning is observed. **(D)** Average intensity plot of fluorescently-labelled EcoRV bound to positively supercoiled DNA (DNA intensity not shown on plot). The position of the Cy5 end label is indicated by a red star. The position of the EcoRV restriction sites are indicated by magenta triangles. **(E)** Average intensity plot of fluorescently-labelled EcoRV bound to negatively supercoiled DNA (DNA intensity not shown on plot). The position of the Cy5 end label is indicated by a red star. The position of the EcoRV restriction sites are indicated by magenta triangles.


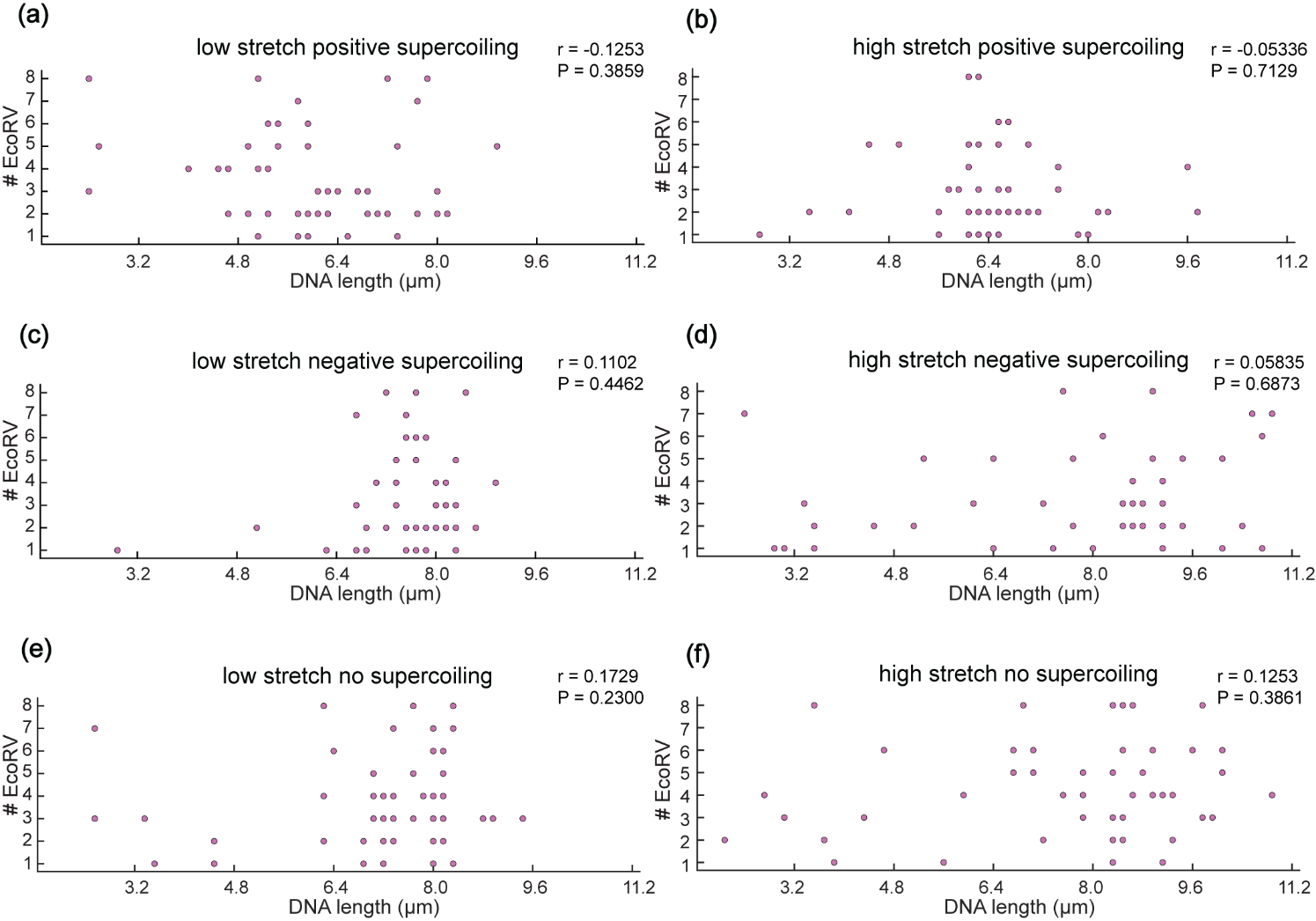


**Supplementary Figure S6:** Scatter plots depicting the relationship between DNA length and the number of EcoRV restriction sites occupied across all conditions, where low stretch and high stretch correspond to high supercoiling conditions and low supercoiling conditions respectively. Spearman’s rank correlation coefficients and p-values are reported to 4 significant figures, and all indicate that there is no significant correlation between DNA length/degree of stretch and EcoRV binding. N = 50 for all conditions individually.


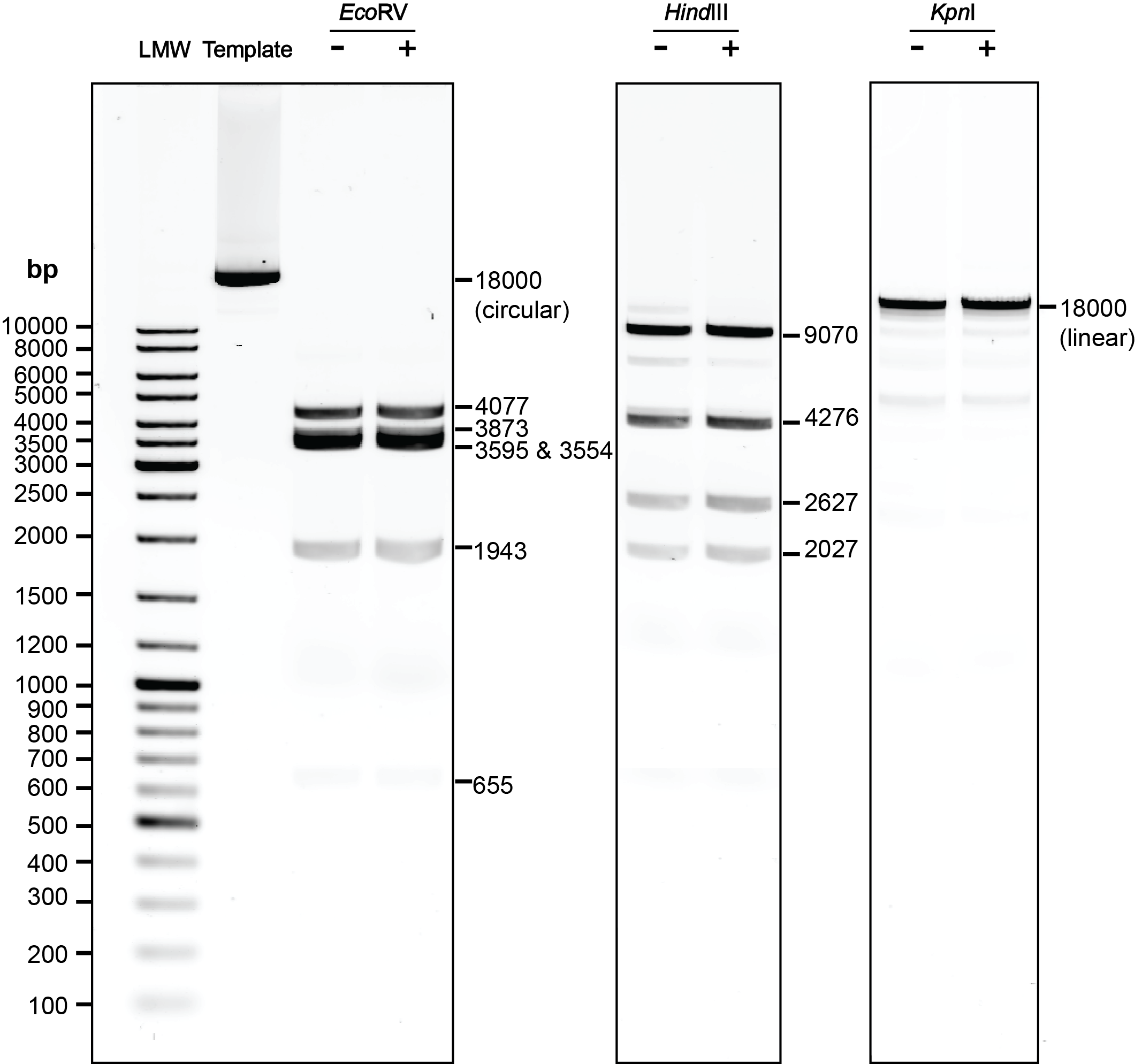


**Supplementary Figure S7:** Gel-based restriction enzyme activity test in the presence and absence of 250 nM SYTOX orange. An 18-kb circular plasmid (pUber) was digested with EcoRV-HF (NEB), HindIII-HF (NEB), and KpnI-HF (NEB) in separate reactions in both the absence (-) and presence (+) of SYTOX orange. All reactions were carried out as per company instructions. Digested products were loaded on a 1% agarose gel to determine the cleavage efficiency of all restriction enzymes. It was determined that all restriction enzymes were fully active, and that SYTOX orange does not influence cleavage activity. See Figure S8 for original uncropped gels.


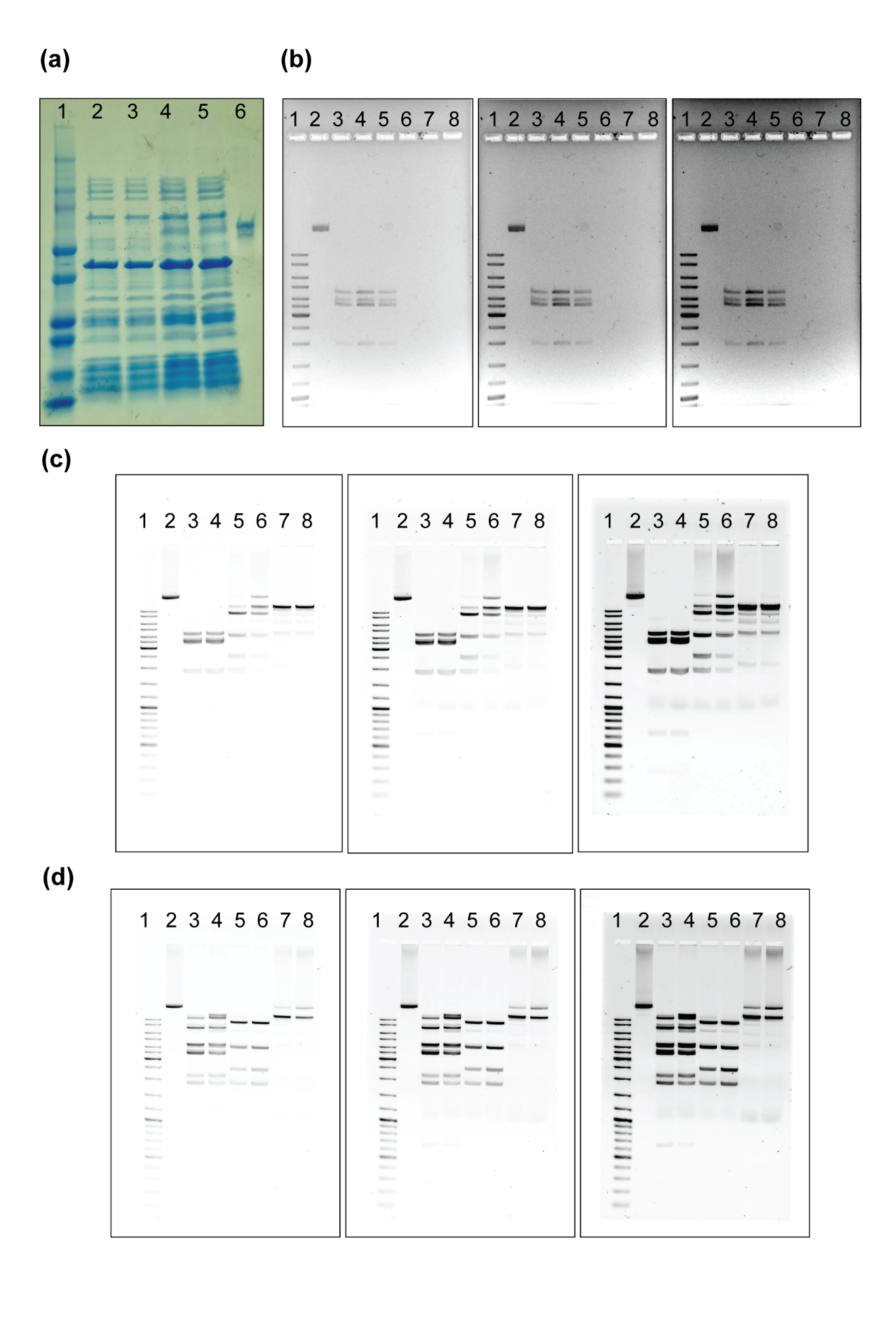


**Supplementary Figure S8:** Original uncropped gels. **(a)** Original SDS-PAGE gel used in Figure S4a. Lanes 1, 2, 4, and 6 were used to make final figure. **(b)** Original agarose gel used in Figure S4b with multiple exposures. Lanes 1, 2, 3, and 4 were used to make final figure. **(c)** Original agarose gel used in Figure S7 with multiple exposures. Lanes 1, 2, 3 ,4, 7, and 8 were used to make the LMW, template, EcoRV -/+ SYTOX Orange, and KpnI -/+ SYTOX Orange lanes respectively. **(d)** Original agarose gel used in Figure S7 with multiple exposures. Lanes 5 and 6 were used to make the HindIII -/+ SYTOX Orange lanes respectively.
